# Do foetal transplant studies continue to be justified in Huntington’s disease?

**DOI:** 10.1042/NS20210019

**Published:** 2021-12-13

**Authors:** Oliver J.M. Bartley, Mariah J. Lelos, William P. Gray, Anne E. Rosser

**Affiliations:** 1 Cardiff University Brain Repair Group, School of Biosciences, Life Sciences Building, Cardiff CF10 3AX, U.K.; 2 Cardiff University Neuroscience and Mental Health Research Institute, Hadyn Ellis Building, Cardiff CF24 4HQ, U.K.; 3 Department of Neurosurgery, University Hospital of Wales Healthcare NHS Trust, Cardiff CF14 4XW, U.K.

**Keywords:** cell replacement, embryonic stem cells, foetal cells, Huntington's disease, induced pluripotent stem cells, transplantation

## Abstract

Early CNS transplantation studies used foetal derived cell products to provide a foundation of evidence for functional recovery in preclinical studies and early clinical trials. However, it was soon recognised that the practical limitations of foetal tissue make it unsuitable for widespread clinical use. Considerable effort has since been directed towards producing target cell phenotypes from pluripotent stem cells (PSCs) instead, and there now exist several publications detailing the differentiation and characterisation of PSC-derived products relevant for transplantation in Huntington’s disease (HD). In light of this progress, we ask if foetal tissue transplantation continues to be justified in HD research. We argue that (i) the extent to which accurately differentiated target cells can presently be produced from PSCs is still unclear, currently making them undesirable for studying wider CNS transplantation issues; (ii) foetal derived cells remain a valuable tool in preclinical research for advancing our understanding of which products produce functional striatal grafts and as a reference to further improve PSC-derived products; and (iii) until PSC-derived products are ready for human trials, it is important to continue using foetal cells to gather clinical evidence that transplantation is a viable option in HD and to use this opportunity to optimise practical parameters (such as trial design, clinical practices, and delivery strategies) to pave the way for future PSC-derived products.

The underpinning concept of cell therapies in regenerative medicine is restoration of structure and function. This can be achieved through several approaches, including implantation of cells to provide support for vulnerable host cells (for example, by releasing key neurotrophic substances) or implantation of cells which will integrate and adopt the function of cells lost to the disease process [1]. Here, we focus on the latter application (replacement of cells lost to the disease process) and consider whether, in light of the development of novel stem cell-derived alternatives, transplants of foetal cells continue to be justified for the neurodegenerative condition, Huntington’s disease (HD).

HD is a devastating neurodegenerative disease which most commonly manifests in mid-life and results in progressive deterioration of movement, cognition and mental health [2]. No disease-modifying treatments are yet available, although there is a global effort to identify such therapeutics [3]. It is an inherited autosomal dominant disorder in which an expansion of >39 CAG repeats in exon 1 of the huntingtin (*HTT*) gene is associated with 100% penetrance. The earliest and most profound pathological change in HD is the dysfunction and degeneration of striatal medium spiny neurons (MSNs), starting years before clinical disease onset [4,5]. This focal loss of a specific cell phenotype makes HD an excellent candidate for regenerative medicine, either alone or combined with other disease-modifying approaches that are being actively explored, such as gene silencing [3].

CNS transplantation has a history going back several decades, with Parkinson’s disease (PD) and HD as the most frequently studied target conditions. Early studies demonstrated the need to transplant immature cells, rather than fully differentiated cells which survive the transplantation process poorly [6]. Selective dissections of foetal brain regions within a specific developmental window can yield cells that have undergone sufficient normal development to be committed to the target cell populations; for HD the relevant foetal brain region is the ganglionic eminence, which gives rise to the striatum and is where MSNs develop [7]. Foetal cells were used to provide key evidence in animal models of HD, that transplanted cells can survive long term in the adult CNS, can continue to mature into appropriate neuronal phenotypes, are capable of integrating into host neural circuitry, and can ameliorate functional deficits (reviewed in [8]). However, it was recognised that in the long-term, clinical application of foetal cells is probably limited as (i) their availability is limited and unpredictable, (ii) they are difficult to transport and store without compromising the tissue quality, requiring a specialised procurement route and facilities, (iii) their preparation is difficult, requiring substantial training and personnel skill as there is little chance to validate their accurate dissection without compromising the final product, and (iv) minimising batch variability between final cell products is limited by uncontrollable factors such as the gestational age, genetic background, the need to sometimes use more than one sample to acquire sufficient cell numbers (not always a requirement for HD where a single sample may suffice) and other issues resulting from variations in the method of acquisition and processing. Overall, these issues make foetal tissue-based cell products highly challenging to use in research/preclinical settings and unsuitable for widespread clinical use [1]. Thus, the need for an alternative cell source that does not suffer these limitations was clear, and considerable effort has since been directed at producing target cells from stem cell populations. For both PD and HD, significant progress has been made in deriving target cell populations from pluripotent stem cells (PSCs) and there are now several publications detailing the production of dopaminergic neurons and MSNs from PSCs, with clinical trials having now commenced for PD using PSC-derived products [9,10].

Considering these advancements, is it still justified to continue pursuing transplants of foetal derived cell products? To determine this, we must consider whether these PSC-derived products are currently a viable alternative to foetal derived cells, particularly with regard to their use in cell replacement therapy. Additionally, we need to consider whether further understanding of the complex developmental course of MSNs is warranted to optimise the novel PSC-derived cell therapy products. For HD, it is currently understood that transplanted cell products will need to reconnect the degenerated neural circuits of the striatum by forming appropriate synaptic connections and performing the function of normal adult MSNs [1]. Therefore, they will likely need to exhibit many aspects of authentic MSNs. It is also likely that the degree of fit to the hosts’ endogenous MSNs will need to be closer than is required for PD, where a cell capable of synaptic release of dopamine into the striatum may be sufficient [11].

There are a number of published protocols for differentiating PSCs towards an MSN-like phenotype, by first inducing a neuroectodermal fate and then guiding regional specificity by manipulating Shh and Wnt pathways [12–17,19,23,25,26] and/or Tgfβ pathways [18,19,23]. Indication of appropriate phenotype is commonly determined by identification of key MSN gene markers such as CTIP2 (BCL11B), FOXP1, and DARPP-32 (PPP1R1B) [20,21], but to date no protocol has been able to consistently produce a high yield of cells expressing these markers. For example, DARPP-32 is present in the majority of mature MSNs in the brain, reported as high as 96% in some studies [22], yet PSC-derived MSN cultures report generally low DARPP-32 yields, often between 10 and 50% of total cells produced [12,14–19,23,26], with only occasional exceptions reporting higher proportions of up to ∼80% [13,25] demonstrating that current methodologies are not yet specific or robust enough to consistently produce a high-yield MSN population. Additionally, these markers are not individually unique to MSNs, nor do they account for the full plethora of biological functions required by normal MSNs. Therefore, expression of these markers alone may not adequately describe an authentic and functional MSN. Indeed, there are data suggesting that such cells are different from *bona fide* MSNs (e.g. foetal derived tissues). Specifically, we have shown that ESC and iPSC derived MSN-like cells have a vastly different epigenome compared with foetal WGE. (i.e. authentic MSN progenitors) [23,24], further indicating that current differentiation protocols do not yet produce cells comparable with genuine MSNs, although single cell analysis is required to more fully understand the functional consequences of such differences.

Despite this, there is some evidence that PSC-derived MSNs may be able to elicit some functional recovery in animal models [13,14,16,25,26], but the mechanisms underlying this recovery are as yet unclear. For example, recovery occurring only 1 month post-graft, when no/few functional synapses have been detected between DARPP-32+ grafted cells and host brain, may suggest trophic support as a mechanism rather than neural circuit reconstruction [26]. There is also some indication that PSC-derived neural cell types that are not pre-patterned to an MSN fate may also be capable of facilitating such recovery, indicating that an MSN-like product may not even be necessary [27–29]. Understanding exactly which features of engrafted cell products are responsible for inducing functional benefits is essential for optimising PSC-derived products further, and critically, determining these factors may be more achievable with authentic foetal tissues because they have undergone a normal developmental processes and have historically provided more consistently functional grafts (for reviews, see [8,30]).

Furthermore, there has been a tendency to focus on single target phenotypes (such as MSNs) and ignore the other neural phenotypes present in foetal donor cell populations (such as glia and interneurons), but the success of foetal transplants may be at least in part due to these additional populations. Indeed, there is now some evidence that transplantation of astrocytes can also offer some functional recovery in models of HD [29,31]. Understanding the role of, and interactions between, the various cells required for optimal transplantations will be easier using a product that already produces a diverse population of high quality, authentic and relevant cells (i.e. human foetal tissue), as opposed to attempting to explore these concepts using multiple challenging and potentially non-optimised PSC protocols and their resulting products. For these same reasons, foetal cells may also serve as the best option for determining other key graft parameters, such as defining the best developmental window to allow optimal cell survival, differentiation and integration post-transplantation. Once the requirements for a viable and functional transplant have been defined, foetal tissues will then act as a useful gold standard to which PSC-derived MSNs can be compared. Thus, we argue that despite the shortcomings of human foetal tissue, it continues to be an important resource for preclinical research aiming to optimise PSC differentiation protocols, cell product composition, transplantation procedures, and serves as a standard by which to evaluate cell products that may have potential for human use.

Given that PSC-derived donor cells are progressing towards, but not currently ready for, clinical trials in HD, we propose that it is important to continue gathering clinical evidence that transplantation is a viable therapeutic option for HD. A number of studies over an extended number of years have provided preliminary data suggesting that the safety of foetal cell transplants is acceptable (reviewed in [32]). There is also preliminary evidence that they can provide functional benefit. Specifically, two pilot studies reported improvements in clinical outcome measures associated with MRI evidence of graft survival, alongside either FDG PET evidence of increased metabolism of the grafted region and frontal cortex (suggesting that the graft had connected to downstream structures), or increased graft region raclopride signal (suggesting at least some MSN-like differentiation in the graft) [33–35]. However, it is also clear that the parameters for successful clinical interventions remain poorly understood and further investigation is required to produce consistently effective outcomes. For example, the Bachoud-Levi et al. went on to undertake the largest foetal tissue transplant clinical trial to date (MIG-HD), which did not meet its primary outcome measures [36]. Notably, there was little evidence of surviving grafts in patients, and no evidence that the cell product was viable at the time of grafting (further discussed in [37,38]). Such outcomes are reflective of the challenges of using foetal tissue, but also demonstrate that large-scale clinical trials investigating cell therapies are a complex process, in need of further refinement [39]. Further clinical studies may therefore represent an important opportunity to optimise the clinical trial parameters (such as trial design and delivery strategies) to pave the way for future trials of PSC-derived products [39].

There remain many challenges to undertaking transplantation studies, irrespective of the donor cell source. One in particular is that of the effective and safe delivery of viable cells. The impermeable nature of the intact blood–brain barrier means that many therapeutics intended to reach the brain cannot be delivered systemically attempts are ongoing to identify viable alternative routes of administration [40]. This is a particular problem for cells where their size and the need for accurate placement means that currently, they need to be delivered directly to the caudate and putamen. Simple scale-up of delivery devices from rodent to human brain have revealed problems of cell sedimentation and cell viability within the much longer and larger bore delivery devices needed to deliver the significantly greater cell numbers needed for human (rather than rodent or non-human primate) brain and the issue of cell reflux back along the delivery needle track [36], emphasising the need for optimisation of delivery routes and devices [41]. Other challenges include achieving optimal targeting of the cell product to the striatum and developing appropriate technical expertise, monitoring to ensure operative fidelity, and regulatory issues around devices and the therapies they deliver for experimental studies. Each of these challenges is surmountable, but they must be addressed alongside optimisation of PSC-derived cell products and can be addressed immediately through well-conducted human studies using foetal cells as the donor source. Despite theoretical concerns around neurosurgical safety [42], the clinical risks of stereotactic neurosurgery are very low and are equally applicable to gene and cell therapy, as the targets and number of tracks required are similar and convection enhanced delivery times for gene therapy delivery are at least as long, if not longer than for cells. Moreover, the practical real-world risks of neurosurgery for gene therapies have been shown to be low and clinically very acceptable and cell delivery is therefore likely to be at least as safe.

The value of exploring cell therapy in HD goes beyond treating individuals with HD; understanding and optimising cell therapy strategies in HD will provide key information for other neurodegenerative conditions. There are several reasons for regarding HD as a model neurodegenerative condition to test cell therapies: it features the major pathophysiological hallmarks of the most prevalent multigenic and/or multifactorial neurodegenerative diseases, including progressive and selective neuronal death, transcriptional dysregulation, mitochondrial dysfunction, and protein aggregation [43]; the range of HD animal models facilitates translation between animal and clinical studies [44]; and the highly reliable genetic and almost complete gene penetrance allows confident clinical diagnosis, greatly increasing power and reliability of clinical studies, compared with conditions in which a definitive diagnosis is more difficult. More specifically, it is an excellent model in which to address the challenges of circuit reconstruction as donor cells are most effective when placed homotopically in their normal position within the striatum, thus allowing restoration of near normal circuitry anatomy [30]. This is in contrast with PD where donor dopaminergic progenitors are grafted ectopically into the striatum, rather than into the substantia nigra (from where their projections are unable to reach and reactivate their normal striatal targets). In this ectopic position they are unable to completely restore normal circuitry. It is also likely that the knowledge and experience gained from cell therapy studies in HD will inform ongoing gene therapy studies and future attempts to achieve endogenous repair.

In summary, we argue that foetal cells remain an essential research tool, offering a precise and detailed understanding of the characteristics of authentic human striatal cells and as a standard against which to test PSC-derived MSNs. We also maintain that human studies of foetal cell transplantation remain important, both for testing the value of cell therapy in HD and for future validation of PSC-derived products in people.

## Data Availability

A data sharing statement is not applicable to the present paper.

## Open Access

Open access for this article was enabled by the participation of Cardiff University in an all-inclusive *Read & Publish* pilot with Portland Press and the Biochemical Society under a transformative agreement with JISC.

## Abbreviations

ESC: Embryonic stem cell
HD: Huntington’s disease
iPSC: induced pluripotent stem cell
MIG-HD: Multicentre Intracerebral Grafting in HD
MSN: medium spiny neuron
PD: Parkinson’s disease
PET: Positron Emission Tomography
PSC: pluripotent stem cell
